# Loss of *PTPRM* Associates with the Pathogenic Development of Colorectal Adenoma-Carcinoma Sequence

**DOI:** 10.1038/srep09633

**Published:** 2015-04-24

**Authors:** Putty-Reddy Sudhir, Shiu-Ting Lin, Chien Chia-Wen, Shung-Haur Yang, Anna Fen-Yau Li, Rai-Hua Lai, Mei-Jung Wang, Yuan-Tsong Chen, Chian-Feng Chen, Yuh-Shan Jou, Jeou-Yuan Chen

**Affiliations:** 1 Institute of Biomedical Sciences, Academia Sinica, Taipei, Taiwan, ROC; 2 Department of Surgery, Taipei Veterans General Hospital, Taiwan, ROC; 3 Department of Pathology, Taipei Veterans General Hospital, Taiwan, ROC; 4 VYM Genome Research Center, Taiwan, ROC; 5 Institute of Genome Sciences, National Yang-Ming University, Taipei, Taiwan, ROC

## Abstract

Identification and functional analysis of genes from genetically altered chromosomal regions would suggest new molecular targets for cancer diagnosis and treatment. Here we performed a genome-wide analysis of chromosomal copy number alterations (CNAs) in matching sets of colon mucosa-adenoma-carcinoma samples using high-throughput oligonucleotide microarray analysis. *In silico* analysis of NCBI GEO and TCGA datasets allowed us to uncover the significantly altered genes (p ≤ 0.001) associated with the identified CNAs. We performed quantitative PCR analysis of the genomic and complementary DNA derived from primary mucosa, adenoma, and carcinoma samples, and confirmed the recurrent loss and down-regulation of *PTPRM* in colon adenomas and carcinomas. Functional characterization demonstrated that PTPRM negatively regulates cell growth and colony formation, whereas loss of *PTPRM* promotes oncogenic cell growth. We further showed that, in accordance to Knudson's two-hit hypothesis, inactivation of *PTPRM* in colon cancer was mainly attributed to loss of heterozygosity and promoter hypermethylation. Taken together, this study demonstrates a putative tumor suppressive role for PTPRM and that genetic and epigenetic alterations of *PTPRM* may contribute to early step of colorectal tumorigenesis.

Genome-wide genetic changes including chromosomal copy number alterations (CNAs) are tightly associated with the development and progression of cancers. To understand the implication of CNAs and altered genes in stepwise progression of colorectal tumorigenesis, a fundamental concept known as adenoma-carcinoma sequence has been considered^1^,^2^,^3^. It has been well demonstrated that mutations or loss of *APC* on chromosome 5q initiates the pathogenic development of most of colon cancer (CRC), followed by subsequent events including gain-of-function mutation of *KRAS* on 12p, loss-of-function of *DCC* on 18q, and loss of tumor suppressor gene *p53* on 17p^1^,^2^,^3^,^4^. To identify additional genetic and epigenetic alterations that promote the pathologic development of CRC, expression profiling as well as comparative genomic hybridization (CGH) have been performed^5^,^6^,^7^,^8^. A cohort of genes have been identified to display differential expression patterns between normal mucosa and adenoma or between adenoma and carcinoma, however, functional roles of these genes remain to be further demonstrated. Chromosomal regions displaying CNAs, including gains on chromosome 7, 8q, 13q, and 20q and losses on 8p, 15q, 17p, and 18q, were also defined to be associated with the progression of colon adenoma to carcinoma. Nevertheless, it remains challenging to extract candidate genes that are responsible for disease progression from the large segments of altered chromosomal regions. By studying the CNAs and gene expression profiles, Carvalho and coworkers have identified *C20orf24, AURKA, RNPC1, TH1L, ADRM1, C20orf20* and *TCFL5* as the putative oncogenes at the amplified region on 20q that are implicated in chromosomal instability-related adenoma to carcinoma progression^9^. Among all these studies, few genes, other than *APC*, have been identified to be involved in the early step of the pathogenic development of CRC.

Protein tyrosine phosphatases (PTPs) are key regulators of protein tyrosine phosphorylation, which controls signaling pathways in regulating cell growth, differentiation, apoptosis, and oncogenic transformation. Loss-of-function events involving receptor type tyrosine phosphatases (PTP) are frequently observed in various types of cancers^10^,^11^,^12^,^13^,^14^. Tyrosine phosphatome analysis has revealed mutations in several PTPs and receptor type PTPs in colorectal cancer^15^. Protein tyrosine phosphatase receptor type M (PTPRM, also named PTP Mu) is a member of the type IIb subfamily of receptor PTPs^16^ and involved in cell-cell adhesion^17^. Decreased expression of PTPRM was demonstrated in breast cancer and associated with poor prognosis^18^,^19^. Loss-of-function of PTPRM was also shown to be a frequent event in acute lymphocytic leukemia due to promoter hypermethylation^18^. The involvement of PTPRM in the pathogenic development of other cancers including CRC remains to be elucidated.

In this study, to further dissect the molecular mechanisms associated with the initiation and progression of CRC, we first identified minimal common regions showing CNAs in adenoma and carcinoma samples by oligonucleotide microarray hybridization. Then we identified significantly altered genes associated with the minimal common regions by means of *in silico* analysis of NCBI GEO colon adenoma or carcinoma expression, and TCGA colon expression and DNA copy number datasets, followed by quantitative PCR analysis. This resulted in the discovery of loss of *PTPRM* on 18p11.2 in the adenoma and carcinoma samples. Functional analysis further supports that loss of *PTPRM* contributes to the pathogenic development of colon adenoma-carcinoma sequence.

## Results

### Genome-wide DNA Copy Number Alterations Associated with Colon Adenomas and Carcinomas

**Figure 1:**
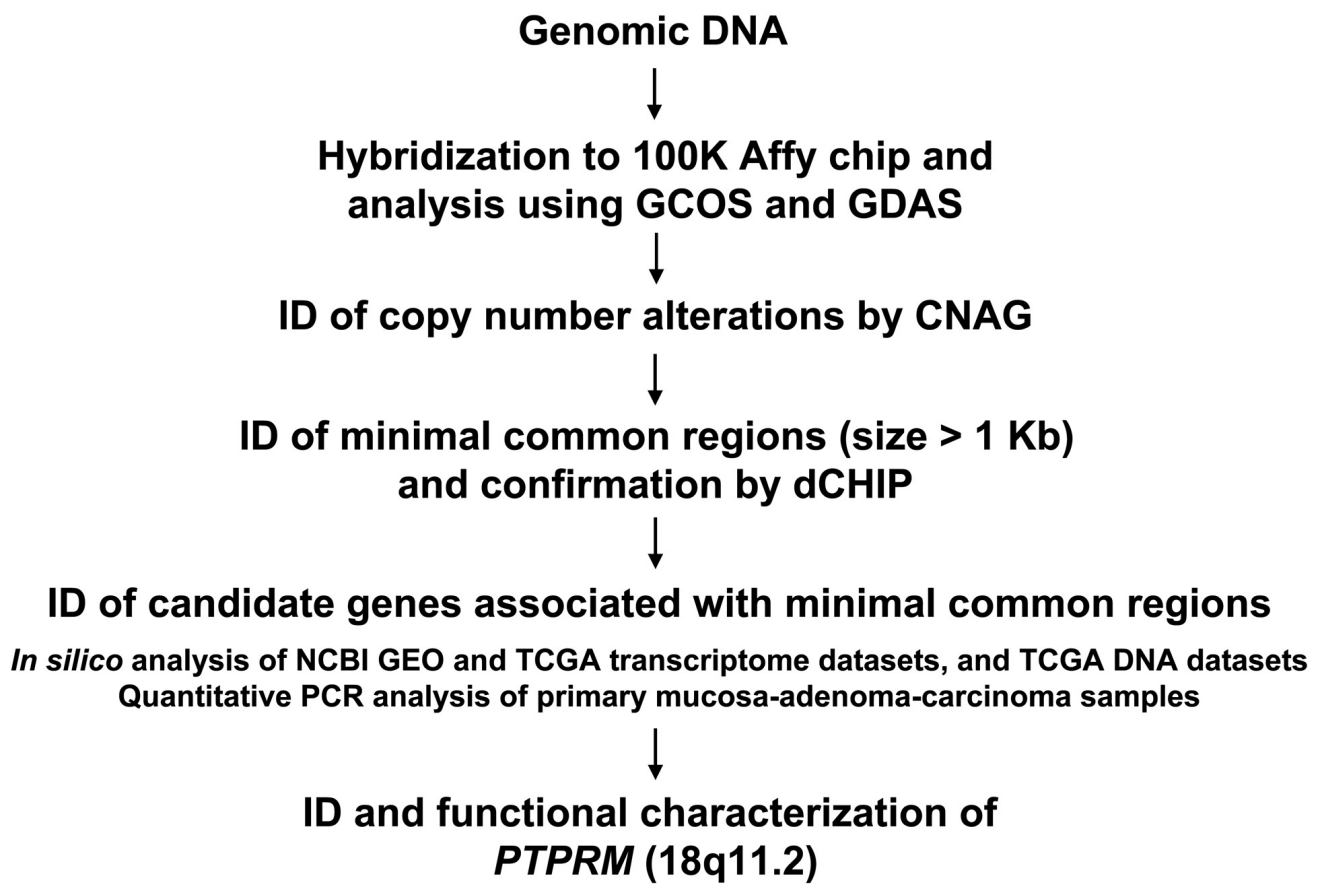
Strategy for identification of CNAs and associated genes in colon adenoma-carcinoma sequence.

**Figure 2:**
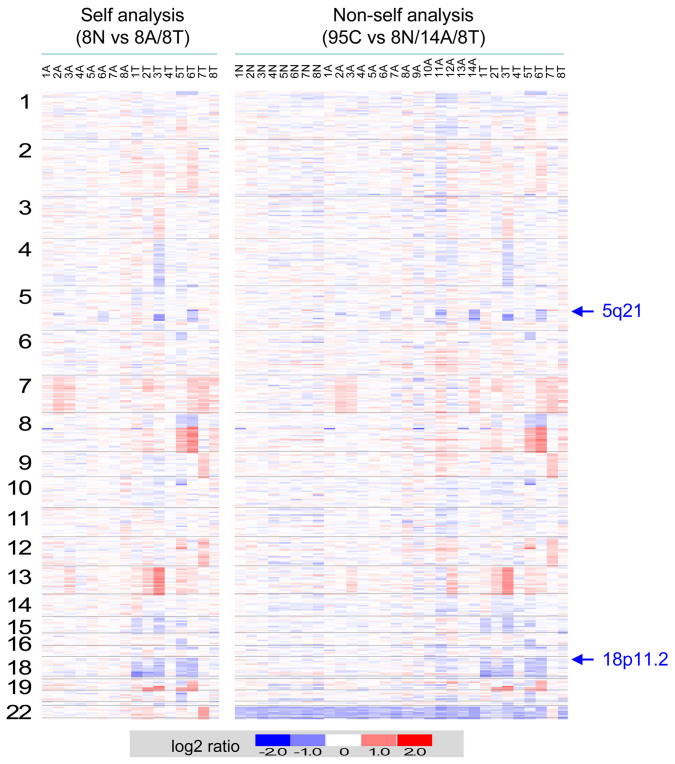
Genome-wide DNA copy-number alterations associated with colon adenoma-carcinoma sequence. Heatmaps of Log_2_ ratio DNA copy number alterations of the adenoma *(A)* and carcinoma *(T)* samples in relation to the non-tumorous mucosa *(N)* by pairwise analyses and to normal control *(C)* by non-pairwise analyses are shown. Chromosomes 1-22 are depicted from top to bottom, and individual samples are shown from left to right. The relative DNA copy number alterations are shown in blue as deletion and red as amplification.

To identify genetic alterations involved in the progressive development of colon cancer, we analyzed CNAs associated with colon adenomatous polyps and carcinomas in comparison to the nontumorous tissues. Genomic DNA was prepared from 8 matching trio-sets of nontumorous mucosa-adenoma-carcinoma samples, and hybridized to high density oligonucleotide array followed by pairwise analyses (8A vs 8N and 8T vs 8N). In addition to the 8 trio-sets of normal mucosa-adenoma-carcinoma samples, 6 additional adenomatous polyps were collected, and the 14 adenomas and 8 tumors were compared to a collection of 95 control references (14A vs 95C and 8T vs 95C) by non-pairwise analyses. Fig. 1 outlines the flowchart of identifying CNAs and associated candidate genes in colon adenoma and carcinoma samples. By CNAG (Copy Number Analyzer for Affymetrix GeneChip Mapping 100K arrays) inspection of chip data, chromosomal regions displaying CNAs in pairwise and non-pairwise analyses were identified. Fig. 2 shows the heatmaps of log2 ratios of DNA CNAs in adenomas and carcinomas by pairwise and non-pairwise analyses. In consistence to previous findings that inactivation of APC gene is an early event while inactivation of DCC is a later event in the multistep genetic processes of colon tumorigenesis, our data showed that loss of 5q21 where the locus of *adenomatous polyposis coli* (APC) resides was found in several adenoma and carcinoma samples, whereas the loss of 18q21 where *Deleted in Colon Cancer (DCC)* locus is located occurred mainly in the carcinoma samples.

Since more adenoma samples were included in the non-pairwise analysis and the majority (92%) of the CNAs observed in pairwise analysis was conserved in those of non-pairwise analysis, we further analyzed the CNAs identified by non-pairwise analysis. In order to reduce the complexity and to conclude significant alterations, minimal common regions with > 1 Kb in size were defined from the identified aberrant regions that were present in at least 2 samples, and such regions were confirmed by dCHIP (DNA-Chip Analyzer) analysis. In total, 96 minimal common regions including 80 CN gains and 16 CN losses were identified (Supplementary Table 1). Candidate gene approach was taken to survey those with size ≤ 3 Mb. Among the 80 regions showing CN gains, 19 regions were identified in both the adenoma and carcinoma samples, and 14 of them were smaller than 3 Mb (Table 1). Among the 16 regions showing copy number loss, a single common loss region of 5.9 Mb size was identified on chromosome 5q21.3-22.3 in both adenomas and carcinomas, and the rest 15 regions were identified in multiple tumor samples, and six regions were of ≤ 3 Mb (Table 1). In total, the 14 gained and 6 lost regions were subjected to further analyses using UCSC genome browser analysis, and 87 genes were identified (Table 1).

### Identification of Critical Candidate Genes Associated with CNAs in Colon Adenoma-Carcinoma Sequence

**Figure 3:**
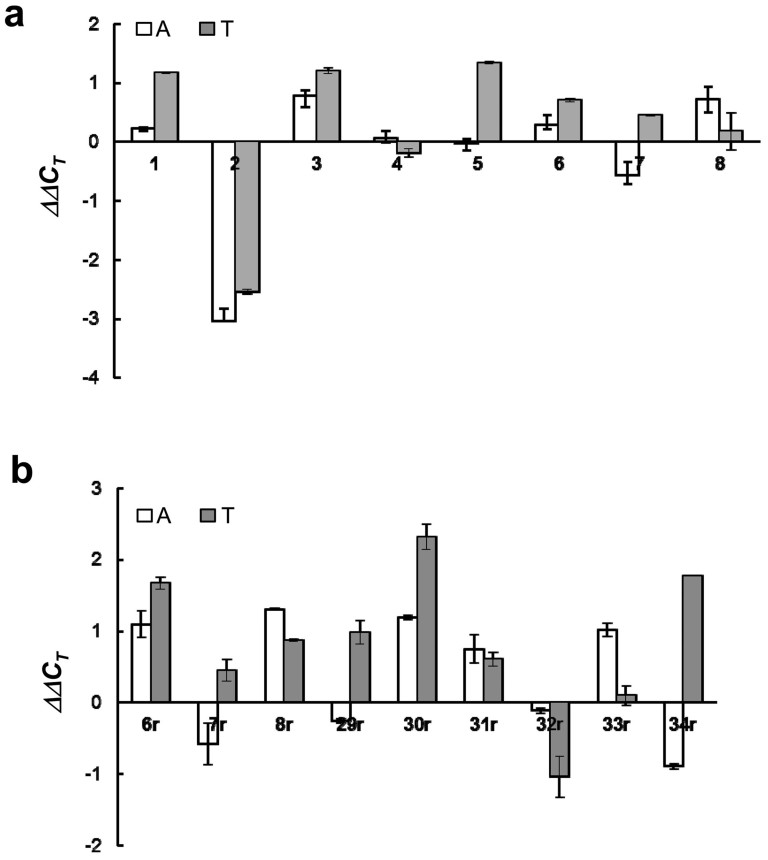
Expression level and copy number alteration of *PTPRM* in primary colon non-tumorous mucosa-adenoma-carcinoma samples. (a) Semi-quantitative PCR analysis of *PTPRM* DNA in 8 trio sets of colon mucosa, adenoma, and carcinoma tissues. Relative levels of genomic DNA and transcripts in adenomatous polyps *(A)* and carcinomas *(T)* are shown as *ΔΔC_T_* in relation to the levels in matching non-tumor mucosa tissue after normalization to the controls, *GAPDH* (for genomic DNA) and *DDX5* (for cDNA). The identification numbers of patients are shown at the bottom of the panel.

To establish the involvement of the candidate genes in the pathogenic development of adenoma polyps and colorectal carcinomas, we first took an *in silico* approach to examine the DNA copy numbers and expression profiles of the candidate genes associated with the 20 minimal common regions (≤ 3 Mb) by means of SMD and NCBI GEO adenoma and carcinoma expression datasets as well as TCGA colorectal cancer DNA and expression datasets available on the Oncomine cancer profiling database (Supplementary Fig. S1). Candidate genes including those exhibited significant copy number changes as well as differential expression patterns (p ≤ 0.001) were selected for further examination. Genes encoding NBN, RIPK2, MATN2, STK3, MED30, KCNK9, DIAPH3, TDRD3, GPC5, ADNP, BCAS4, CEBPB, PARD6B, RNF114, SNAI1, PTPRM, DSC2, and CECR1 were subjected to semi-quantitative PCR analyses of the changes in DNA copy number and transcript expression level during the pathogenic development of normal mucosa-adenoma-carcinoma sequence. In consistence to the CN loss of *PTPRM* locus revealed by *in silico* approach, PCR analysis revealed that 25% (2 of 8) adenomas and 50% (4 of 8) carcinomas displayed CN loss of PTPRM locus (Fig. 3A). Quantitative RT-PCR analyses of an additional set of 9 trios of non-tumor-adenoma-carcinoma samples also showed decreased expression of *PTPRM* in 56% (5 of 9) adenoma and 67% (6 of 9) carcinomas (Fig. 3B). Our data showed that down-regulation of *PTPRM* was a frequent event in the colorectal adenomas and carcinomas. We further examined the significance of PTPRM loss using large number of samples by analyzing 26 colorectal datasets, including 5 adenoma and 21 carcinoma datasets, which are available in Oncomine database. The Oncomine-based data analysis revealed frequent down-regulation of PTPRM in both adenoma and carcinoma samples (Supplementary Fig. S2). Therefore, *PTPRM* was further characterized for its functional role in colon tumorigenesis.

### Expression of PTPRM Suppresses Tumor Cell Growth and Colony Formation

**Figure 4:**
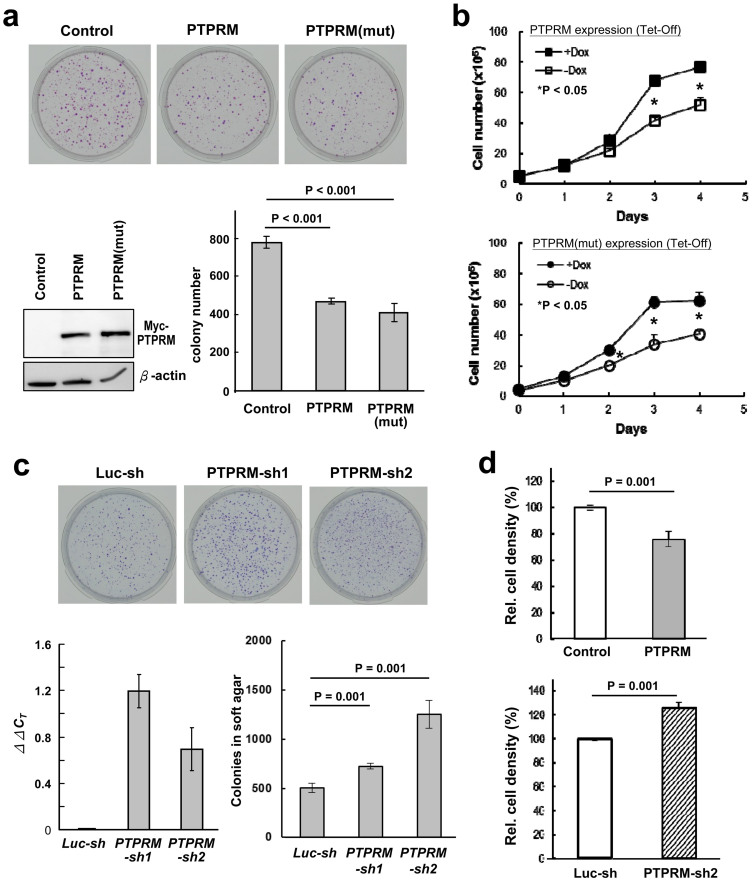
Ectopic expression of PTPRM suppresses cell growth and colony formation, whereas knockdown of *PTPRM* promotes anchorage-independent cell growth. (a) Ectopic expression of PTPRM suppresses colony formation. HCT116 cells were transfected with vectors containing the full-length cDNA of PTPRM in right (PTPRM) and reverse (Control) orientations as well as the PTP domain mutant (PTPRM(mut)), and cultured in neomycin-containing medium. Colonies were counted after two weeks and presented in bar graph. Western blot shows the expression of PTPRM. (b) PTPRM suppresses cell growth independent of its phosphatase activity. Tet-Off HeLa cell clones stably harboring PTPRM cDNA (Top panel, the wild-type PTPRM; Bottom panel, PTPRM mutant) were selected, and cultured in medium containing G418, puromycin and doxycycline. For cell growth assay, cells were incubated with fresh medium without doxycycline for 48 hrs to induce the expression of PTPRM, and cell numbers were counted using CCK-8 reagent at indicated time. (c) Knockdown of *PTPRM* promotes anchorage-independent growth in soft agar. Soft agar colony formation assay was performed in colon cancer SW620 cell clones stably harboring lentivirus-encoded control shRNA or shRNA targeting *PTPRM* as indicated. Two individual shRNAs targeting at *PTPRM* were employed. Expression of *PTPRM* transcripts in the cell clones harboring the individual shRNAs was measured by semi-quantitative RT-PCR analysis. *ΔΔC_T_* was derived by comparing the *C_T_* of the *PTPRM-KD* clones to that of the *luciferase-KD* clone after normalization to GAPDH. Data shown are means ± SD of three independent experiments. *P < 0.05 by Student's t test. (d) Saturation density was measured in the Tet-Off HeLa cell clone expressing PTPRM after removal of doxycycline (Top) and SW620/Luc-KD and PTPRM-KD clones (Bottom). Cells were cultured, with fresh medium changed every two days till reaching confluency. Cells were changed into fresh medium and cell numbers were counted by CCK-8 assay after culturing for two more days.

To examine the role of *PTPRM* in colon tumorigenesis, gain-of-function and loss-of-function approaches were employed. We first introduced an expression vector encoding *PTPRM* into HCT116 cells which expressed endogenous *PTPRM* at modest level, and monitored the effect of ectopic expression of *PTPRM* on cell growth. As shown in Fig. 4A, transient expression of PTPRM yielded reduced number of colonies relative to the control plasmid. To ascertain the growth suppressive activity of PTPRM, we generated HeLa cell clones overexpressing ectopic PTPRM in an inducible Tet-Off system. As shown in Fig. 4B, induction of PTPRM expression in the premade Tet-Off Hela cells by removing deoxycyclin led to decreased cell growth by MTT assay. In addition, we further examined whether the phosphatase activity is a prerequisite for the suppressive activity of PTPRM by introducing mutations to the critical cysteine residue in the PTP signature motif C(X)_5_R of both PTP domains. As shown, inducible expression of the PTPRM mutant in which the Cys^1095^ and Cys^1389^ residues in the PTP signature motifs were changed into serine also suppressed cell growth, suggesting that phosphatase activity is not critical for PTPRM-mediated suppressive activity. In consistence, transient expression of PTPRM mutant also suppressed colony formation (Fig. 4A). We also performed loss-of-function study to knock down the expression of endogenous *PTPRM* in colon cancer SW620 cells using two independent sh-RNAs that targeted at *PTPRM* at different regions, and investigated whether loss of *PTPRM* would contribute to cell transformation. Expression of PTPRM was monitored by qRT-PCR due to the lack of good PTPRM-specific antibodies. As shown, introduction of two independent *PTPRM-shRNAs* led to decreased expression of *PTPRM* transcripts (Fig. 4C, lower left panel) and increased numbers of colonies formed in soft agar (Fig. 4C, lower right panel), indicating that loss of *PTPRM* promotes anchorage-independent cell growth in soft agar. In consistence to that PTPRM suppressed cell growth and knockdown of *PTPRM* promoted colony formation in soft agar, we also showed that induction of PTPRM expression in HeLa cells resulted to a reduced saturation density, whereas knockdown of endogenous PTPRM expression in SW620 cells led to an increased saturation density (Fig. 4D). All these data suggest that PTPRM negatively regulate cell growth, and knockdown of PTPRM promote oncogenic activity.

Next, we looked into the deregulation of PTPRM-associated pathways in colorectal cancer by means of Broad Institute Gene Set Enrichment Analysis (GSEA) tool^20^ (http://www.broad.mit.edu/gsea/). Three PTPRM-associated pathways, nectin pathway, adherens junction, and cell adhesion molecules CAMs, were obtained from molecular signature database^21^ of GSEA. We analyzed the expression datasets of colon adenocarcinoma and rectal adenocarcinoma, which are available on TCGA database. GSEA analysis revealed that gene sets related to adherens junction pathway and cell adhesion molecules pathway displayed statistically significant and concordant differences in their expression pattern in rectal adenocarcinoma (Supplementary Fig. S3). This pathway analysis may aid to probe the role of PTPRM in the initiation and progression of colorectal cancer pathogenesis.

### Inactivation of PTPRM by Genetic and Epigenetic Alterations

**Figure 5:**
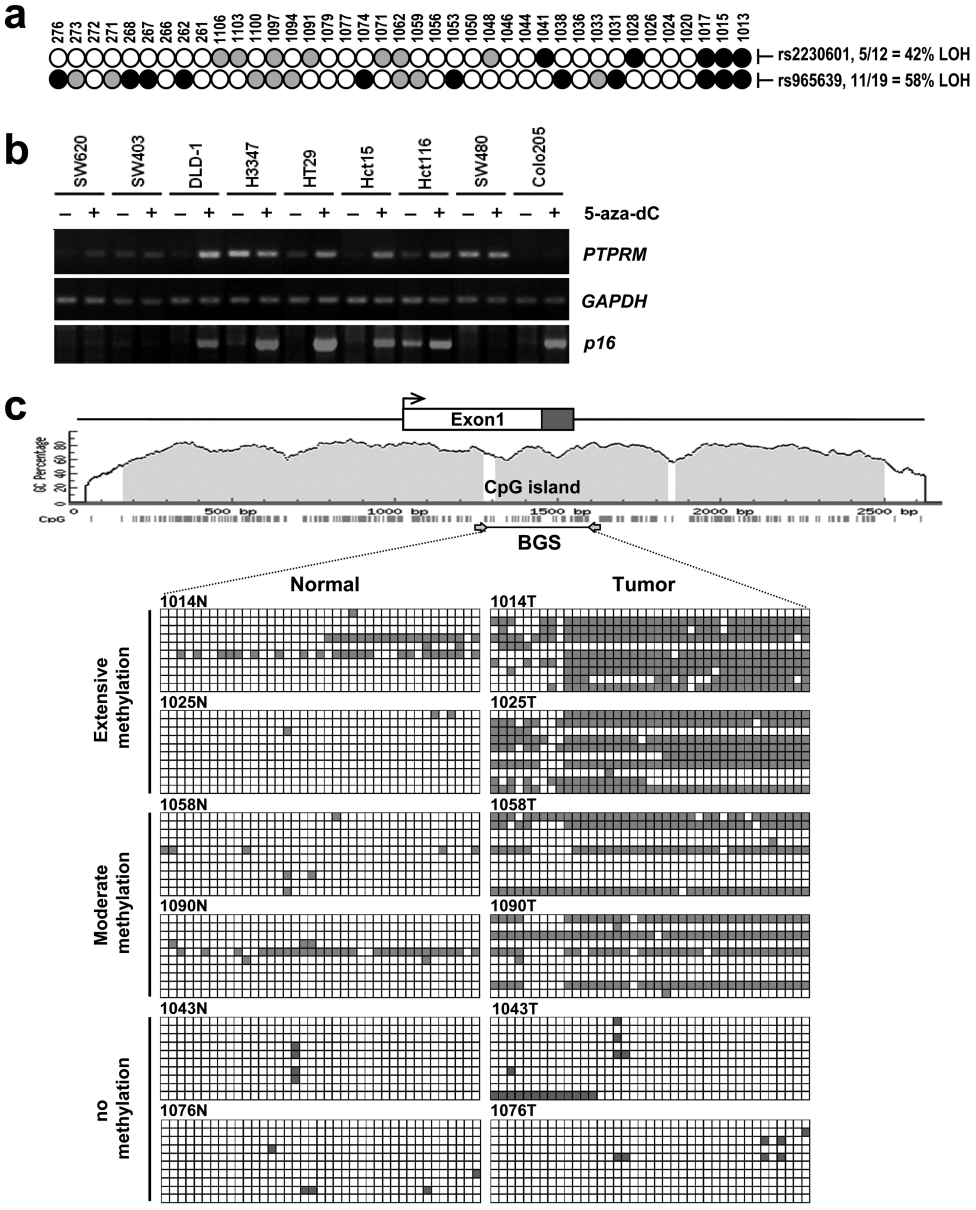
Genetic and epigenetic alterations of *PTPRM* in colorectal cancer. (a) Assessment of LOH of PTPRM locus on chromosome 18p11.2 in CRC. Two PRPRM gene-specific SNP markers, rs2230601 and rs965639, were assessed for LOH (closed circle), retention of heterozygosity (gray colored circle), or non-informative (open circles) in 39 primary CRC samples. The frequency of LOH for each marker in the informative cases is shown at right. (b) RNA was prepared from nine CRC cell lines treated with (+) and without (-) 5-aza-dC and subjected to RT-PCR analyses of the expression of PTPRM, p16 and GAPDH transcripts. (c) Bisulfite sequencing analysis of methylation of PTPRM promoter in primary colorectal carcinoma and corresponding mucosa samples. The genomic structure of PTPRM and the relative position of CpG island are depicted. Exon 1 is shown as the open box, with coding region shown in the shaded area. An enlarged representation of the CpG island is sketched as a solid line with relative positions of the CpG sites depicted as short vertical lines. Bisulfite-modified genomic DNA was amplified by PCR using primer set indicated, PCR products were subcloned, and ten isolates were sequenced for each sample. The CpG sites examined are shown in open (for unmethylated) and filled (for methylated) squares. Tumors displaying methylated CpG sites in at least 5 clones were designated as extensively methylated, 2-5 clones as moderately methylated, and scattered methylation as unmethylated. Representative tumors from each category are shown.

We further examined the mechanisms undermining the inactivation of *PTPRM* in CRC. Loss of heterozygosity (LOH) assay was performed to determine the frequency of allelic loss of *PTPRM* locus using single nucleotide polymorphism (SNP) markers *rs2230601* and *rs965639*, which are located at the exon 8 and intron 23 of *PTPRM* gene, respectively. In consistence to the high-density oligonucleotide array data showing CN loss in the region harboring *PTPRM* locus in colorectal carcinomas, high percentage of LOH was observed. In 39 pairs of primary CRC and their corresponding non-tumorous samples examined, LOH of *rs2230601* and *rs965639* was detected in 42% (5/12) and 58% (11/19) of informative cases (Fig. 5A), respectively. These data suggest that LOH is a frequent genetic event leading to the inactivation of *PTPRM* in CRC.

To fulfill Knudson's two-hit hypothesis, we examined whether promoter hypermethylation would account for an additional hit to inactivate *PTPRM* in CRC. First, we measured the expression of *PTPRM* in colon cancer-derived cell lines cultured in the presence and absence of 5-aza-2′-deoxycytidine (5-aza-dC), a methyltransferase inhibitor. As shown in Fig. 5B, 5-aza-dC treatment led to an increased expression of *PTPRM* in 5 of 9 cell lines examined. In parallel, the expression of *p16*, which is known to be regulated through promoter hypermethylation, was significantly up-regulated upon 5-aza-dC treatment in 6 of the 9 cell lines, whereas the expression of house-keeping gene, *GAPDH* remained unchanged. A putative CpG island was identified at the 5′-region of the *PTPRM* gene spanning from the promoter, exon 1 to intron 1 (Fig. 5C). Bisulfite sequencing analysis was performed to examine the methylation status in a pre-determined representative region in 26 of the 39 primary colorectal carcinomas and their corresponding non-tumor tissues. Extensive and moderate hypermethylation of *PTPRM* promoter was detected in 15.3% (4/26) and 15.3% (4/26) tumors, respectively. Aberrant methylation of *PTPRM* promoter was detected mainly in the tumor, as the matching non-tumor mucosal tissues only showed scattered methylation in *PTPRM* promoter region. These data suggest the expression of *PTPRM* is regulated by epigenetic modification through promoter hypermethylation in a subset of CRC. Taking together, our data suggest that LOH and epigenetic modification through promoter methylation may contribute to the inactivation of *PTPRM* in CRC.

## Discussion

Intensive research on colorectal cancer has revealed various driver genes involved in this disease by means of genetic and genomics approaches. Examining the CNAs in colorectal adenoma-carcinoma sequence may shed light on the genetic events associated with the initiation and progression of CRC. In this study, we have adopted a global approach using high-throughput oligonucleotide microarray (GeneChip Mapping 100K set) followed by expression profile evaluation to identify CNA-associated genes involved in colon tumorigenesis. In line with previous findings that deletion of 18p is a frequent event in CRC^22^, our study showed recurrent loss of 18p11, where the *PTPRM* locus resides, in the carcinoma lesions by oligonucleotide microarray. By *in silico* and qPCR analyses, we further demonstrated that the gene and transcript copy numbers corresponding to *PTPRM* were significantly decreased in both primary colon adenomas and carcinomas in comparison to the non-tumorous tissues. We noted that hybridization-based microarray analysis displayed lower sensitivity in detecting CN loss and should be assisted by *in silico* and/or qPCR analysis in searching for genes that are involved in loss-of-function event. Since loss of *PTPRM* locus was a common event in both colon adenomas and carcinomas, functional characterization was further performed. Our data showed that ectopic expression of *PTPRM* suppressed tumor cell growth, whereas depletion of *PTPRM* facilitated anchorage-independent cell growth and increased saturation density. In agreement to Knudson's two-hit hypothesis, we further showed that *PTPRM* is frequently inactivated in CRC through LOH and promoter hypermethylation. These data suggest a tumor suppressive role of PTPRM and that loss of *PTPRM* may promote oncogenic transformation.

By turning off growth factor- and cytokine-mediated signaling pathways, PTPs play an important role in regulating a variety of cellular processes including cell growth, differentiation, mitotic cycle, and oncogenic transformation. De-regulation of PTP activity is frequently associated with cancer development and progression^23^,^24^. Loss-of-function of receptor type PTPs has been frequently observed in various types of cancer. This work and several other microarray studies have demonstrated down-regulation of *PTPRM* in colon adenomatous and tumorous samples. LOH of *PTPRJ*, encoding another member of the receptor type PTP family proteins, was reported to be an early step in colon cancer^25^. Aberrant DNA promotor hypermethylation of *PTPRG*, *PTPRK*, *PTPRM* and *PTPRO* genes has been shown to be associated with acute lymphoblastic leukemia^18^. These results suggest that inactivation of receptor type PTPs including PTPRM may promote the pathogenic development of human cancers.

Our data that PTPRM negatively regulates cell growth suggest a tumor suppressive role of PTPRM. The fact that we were unable to recover neomycin-resistant cell clones stably expressing PTPRM in long-term culture also agrees with its role as a tumor suppressor. PTPRM belongs to the type IIb subfamily of receptor type PTPs. It has a large extracellular region which contains a MAM (meprin/A5-protein/PTP mu) domain, an immunoglobulin-like (Ig) domain, and four fibronectin type III (FNIII) repeats. PTPRM has been shown to mediate cell-cell aggregation^26^,^27^. PTPRM facilitates cell-cell interaction in a homophilic manner via the binding of the MAM and Ig domains to the FN1 and 2 domains of another molecule on an adjacent cell, which is independent of phosphatase activity^27^,^28^. In our study, we showed that disruption of PTPRM phosphatase activity by introducing the mutations in the cysteine residues conserved in both PTP domains did not show significant effect on its suppressive activity, suggesting the possible roles of extracellular domains of PTPRM in PTPRM-mediated tumor suppressor activity through cell-cell interactions. In support, pathway analysis using GSEA tool revealed the deregulation of the PTPRM-associated pathways including cell adhesion molecules pathway and adherens junction pathway. Further studies on the characterization of the functions of PTPRM extracellular domains in the regulation of cell-cell adhesion and the dissection of how the hemophilic interactions trigger signaling to regulate cell homeostasis may aid to reveal the important role of PTPRM in colon cancer development and progression.

We report here, for the first time, the functional implication of *PTPRM* loss in colorectal tumorigenesis. The tumor suppressor role of PTPRM was demonstrated by its ability to suppress colony formation. In agreement to its tumor suppressive role, loss of function of PTPRM in colon cancer was achieved through LOH and promotor hypermethylation. We believe that the data reported on CNAs as well as the functional role of candidate gene *PTPRM* involved would be helpful for the better understanding of the mechanisms involved in colorectal tumorigenesis.

## Methods

### Study Subjects, DNA and cDNA Preparation

Genomic DNA was extracted from 125 samples for oligonucleotide microarray analysis: 8 complete sets of non-tumorous mucosa, adenomatous polyps, and carcinoma tissues (1N - 8N, 1A - 8A, & 1T - 8T) obtained from colorectum of colon cancer patients, additional 6 polyps samples (9A - 14A) from the adenoma patients, and mononuclear peripheral blood samples from 95 healthy individuals (95C). Genomic DNA from normal mucosa was used as self reference for the corresponding polyps and tumor samples. The mononuclear peripheral blood samples from the healthy individuals served as the non-self reference for the total 14 polyps and 8 tumor samples. In addition to the above mentioned samples, cDNA from nine trios of normal mucosa-polyps-carcinoma samples were studied by semi-quantitative PCR. In addition, genomic DNA was prepared from the tumor and the corresponding non-tumor samples of 39 colon cancer patients for loss of heterozygosity assessment and promoter hypermethylation study.

Genomic DNA was isolated by standard proteinase-K digestion and phenol/chloroform extraction procedure^29^. Total RNA was prepared from homogenized tissues, converted to cDNA and subjected to polymerase chain reaction (PCR) as described^30^. Immunohistochemistry was performed with 5-micrometer thick sections of patients' achieves as described previously^31^.

### DNA Copy Number Analysis

Genome-wide copy number analysis was performed using Affymetrix 100 K SNP array according to the manufacturer's instructions. In brief, 250 ng of genomic DNA from each sample was subjected to digestion with two individual restriction enzymes (*Hind*III or *Xba*I). The digested fragments were ligated and amplified by means of PCR where the conditions were set up to amplify the fragments with 250–2000 bp length. After amplification, 40 μg of PCR product was purified and fragmented to an average size of less than 180 bp extent. The fragmented DNA was subjected to biotin labeling and hybridized to the GeneChip mapping 100 K microarray for 16 hr. Then the array was washed and stained for 2 hr and scanned with GeneChip Scanner and the data was analyzed by CNAG and dCHIP algorithms.

The GeneChip scanner produced DAT (raw image file) files were collected and extracted by GeneChip operating software (GCOS), which converts the data to the specific intensity probe files (CEL files). GeneChip Geneotyping analysis software GTYPE (previously known as GDAS) is useful for further analysis of data stored in the GCOS software. By using CDF library files, GTYPE converts the data to CHP files, which contain summary information of probe sets including intensity values. In this study, CNAG, version 2.0 (Copy Number Analyzer for Genechip^32^) and dCHIP (DNA chip^33^), version 2007 were used to analyze copy number changes. Both 50 K Xba240 and 50 K Hind240 arrays were combined for the analysis of CNVs. CNAG software is able to read the CHP whereas dCHIP can read CEL and TXT files for the analysis of copy number changes. The best fit reference default option (number 10) was utilized in CNAG analysis to reduce the noise. However, in dCHIP we used 95C as non-self reference set. Copy number changes were analyzed from average intensity of 5 consecutive SNPs using CNAG and dCHIP. Finally, the regions (hg17) with copy number changes were listed and the genes from corresponding regions (hg19) were identified using UCSC Genome Browser.

### Semi-quantitative PCR

Semi-quantitative PCR was performed on an MJ Research DNA Engine Opticon 2 system using FastStart SYBR Green I. Transcripts of 12 genes were measured. The primers used for amplifying the cDNA of *PTPRM* and two control (*GAPDH* and *DDX5*) are listed: *PTPRM* (NM_001105244), sense primer 5′-CTG CCT CTT TGA TGA GCC GTA T-3′, antisense primer 5′-CAG AAG TCG GTT TAG TCA AGG TGT T-3′; *GAPDH* (NM_002046), sense primer, 5′-TGC CAA ATA TGA TGA CAT CAG AAG-3′; and antisense primer, 5′- GTG GGT GTC GCT GTT GAA GTC-3′; and *DDX5* (NM_004396), sense primer 5′-CAG ACT GGA TCT GGG AAA ACA-3′, antisense primer, 5′-GTG CCA GCA CCA AAC AAA T-3′. Same sets of primers were used for amplifying *PTPRM* and *GAPDH* in genomic DNA. The threshold cycles (*C*_T_) is defined as the fractional cycle number at which the fluorescence passes the fixed threshold and was recorded for all samples for both the target gene and the reference gene. Melt curve analysis was performed for each run. Relative gene expression or gene copy number of the target gene was calculated as Δ*C*_T_, determined by subtracting the *C*_T_ of reference gene from the *C*_T_ of target gene. Relative differential expression or copy number of the target gene in the tumor and adenoma samples was shown as ΔΔ*C*_T_, determined by subtracting the Δ*C*_T_ of the tumor and adenoma samples from the Δ*C*_T_ of its nontumorous mucosal sample.

### TaqMan SNP Genotyping Assay

Genomic DNA isolated from CRC and the matching non-tumor tissues from 39 patients was subjected to loss of heterozygosity assessment using TaqMan SNP Genotyping Assays on an Applied Biosystems 7900HT Sequence Detection System (Foster City, CA, USA). SNP markers rs2230601 and rs965639, located at exon 14 and intron 33 of *PTPRM* gene, respectively, were studied. The minor allele frequency of SNP rs2230601 and SNP rs965639 was estimated as 0.244 and 0.356 in Hans populations, respectively (NCBI SNP database). Results were collected and analyzed by SDS version 2.2 software (Applied Biosystems). A sample showing both alleles (AB) in the normal and only one allele (AA or BB) in the tumor duplicates was scored as LOH.

### Genomic Sequencing of Bisulfite-modified DNA

Using UCSC Genome Browser program, a CpG island of *PTPRM* gene was identified. The methylation status of a representative region of the CpG island was determined by genomic sequencing of bisulfite-modified DNA as described^10^,^34^. The bisulfite-modified DNA was subjected to PCR amplification using AmpliTaq Gold DNA Polymerase (Applied Biosystems) and the primers (forward primer, 5′-ATA GGG GAG GAG GAT TTA GGA TT-3′, and reverse primer, 5′-AAA ACT CTA CCA CCA AAA AAC TAT C-3′). PCR products were gel-purified, cloned into pGEM-T-easy vector (Promega, San Luis Obispo, CA, USA), and ten clones were arbitrarily selected for automatic fluorescence-based DNA sequencing.

### 5-Aza-2′-deoxycytidine Treatment

Cells were seeded in complete medium at a density of 5 × 10^5^ cells in 6-cm dishes. After 12 h, cells were treated with 5 µM 5-aza-2′-deoxycytidine (Sigma, St Louis, MO, USA). After 4 days, cells were harvested and analyzed.

### Constructs

PTPRM expression vector was generated by PCR amplification of the full-length cDNA of PTPRM using EST clone BC151842 as template in the presence of gene-specific primers (forward primer: PTPRM-F(Xho), 5′-CTCGAGATGAGGGGACTTGGGACTTGC-3′; reverse primer: PTPRM-R(HindIII), 5′-AAGCTTAAGCCAGAATTCAAGTATTCCAG-3′) followed by cloning the PCR product into pGEM-T vector, and subsequent subcloning the insert into pcDNA3.1, yielding pcDNA3.1-PTPRM-Myc-His plasmid. Expression vector encoding PTPRM(C1095S/C1389S) was constructed by two consecutive 2-step PCR cloning processes. PTPRM-C1095S mutant was initially generated. In the 1^st^ PCR, the 5′- and 3′-cDNA fragments corresponding to the regions of amino acids 1-1095S and amino acids 1095S to 1452 were individually amplified using pcDNA3.1-PTPRM-Myc-His plasmid as template in the presence of designated primers. (5′-fragment: forward primer, PTPRM-F(Xho); reverse primer, C1095S-R, 5′-ACCAGCACTGCTGTGCACCACCAG-3′; 3′-fragment: forward primer, C1095S-F, 5′-GTGGTGCACAGCAGTGCTGGTGCA-3′; reverse primer, PTPRM-R(HindIII)). The PCR products from the 1^st^-step reactions were combined and served as templates for 2^nd^-step PCR using primers flanking the full-length cDNA (forward primer, PTPRM-F(Xho); reverse primer, PTPRM-R(HindIII)). The PCR product was subcloned as described above to yield pcDNA3.1-PTPRM(C1095S)-Myc-His plasmid. Then the 2-step PCR was repeated one more time using specific primers and pcDNA3.1-PTPRM(C1095S)-Myc-His as template to generate pcDNA3.1-PTPRM(C1095S/C1389S)-Myc-His plasmid. In the 1^st^ PCR, the 5′-cDNA fragment (corresponding to amino acids 1-1389S) and the 3′-fragment (corresponding to amino acids (1389S to 1452) were individually amplified using mutant-specific primers (5′-fragment: PTPRM-F(Xho); reverse primer, C1389S-R

5′-CCCGTTCAAGCTGTGCACAACCGT-3′; 3′-fragment: forward primer, C1389S-F

5′-GTTGTGCACAGCTTGAACGGGGGA-3′; reverse primer: PTPRM-R(HindIII)). The PCR products were combined to serve as template for the 2^nd^-step PCR as described above. Both the pcDNA3.1-PTPRM-Myc-His and pcDNA3.1-PTPRM(C1095S/C1389S)-Myc-His, designated as pcDNA3.1-PTPRM(mut)-Myc-His in this study, were sequence-verified by sequencing analysis.

### shRNA Knockdown Experiment

Lentiviral pLKO.1 vector harboring shRNA against PTPRM was purchased from National RNAi Core Facility, Taiwan. Two shRNAs targeting PTPRM were employed (*PTPRM-sh1*, target sequence: 5′-CGACGCTTCATTGCTTCATTT-3′; *PTPRM-sh2*, target sequence: 5′-GAGTCGTCGTTTCTGTCAGAT-3′). Briefly, individual pLKO.1-shRNA plasmid was cotransfected with packaging plasmids pMD.G and pCMVΔR8.91 into 293T cells by LF2000. After 48 hrs, medium was collected for virus preparation. To knockdown the expression of PTPRM, SW620 cells were infected with lentivirus (at MOI~5), cells were selected by puromycin for 14 days.

### Colony Formation Assay

For colony formation assay, HCT116 cells were transfected with pcDNA3.1-PTPRM-Myc-His or the same vector containing the 4.5-kilobase long full-length PTPRM cDNA inserted in reverse orientation as a control to monitor the transfection efficiency by LF2000 (Life technology). Cells were cultured in the presence of G418, with medium replaced with fresh medium every two days. After 12 days, cells were stained with crystal violet and the number of colonies was counted by ImageJ software.

Alternatively, soft agar assay for anchorage-independent cell growth was performed. A total of 2 × 10^4^ SW620 cell clones stably harboring PTPRM-shRNAs or the control Luciferase-shRNA were resuspended in 3 mL of 0.3% soft agar in culture medium and overlaid on 3 mL of 0.5% plating agar in 6-cm dish. Cells were cultured for 3 weeks by replacing the medium every 3 days. Cells were stained with 0.005% Crystal violet and the plates were air-dried. Images were taken and colony number and size were counted by ImageJ software.

### Inducible expression of PTPRM and Cell Proliferation Assay

To express PTPRM under an inducible system, the cDNA encoding the wildtype and the mutant PTPRM was further subcloned into pTRE2pur vector. The pTRE-PTPRM and pTRE-PTPRM(mut) plasmids were individually transfected into the premade Tet-Off HeLa cells, and cultured in the presence of G418, puromycin and doxycycline for 21 days. Single colonies were selected and maintained in the medium containing G418 (100 μg/ml), puromycin (0.5 μg/ml) and doxycycline (1 μg/ml). For inducible expression of PTPRM, cells were incubated with fresh medium without doxycycline for 48 hrs prior to further experiments. For cell proliferation assay, medium was changed with fresh medium every 2 days, and cell numbers were counted using Cell Counting Kit-8 reagent (Sigma) at indicated time.

## Supplementary Material

Supplementary Information Supporting information

## Figures and Tables

**Table 1:** Regions of copy number alterations and candidate genes associated with colon carcinomas. CNAs were identified by 100K oligonucleotide array analysis, and regions that are ≤ 3 Mb in size are analyzed. Candidate genes are identified using UCSD genome browser. CN gains (+), CN loss (-), cytoband, physical position are shown

	Cytoband	Common region	Genes involved
+	8q21.3	90,709,838-92,033,734	NECAB1, CALB1, DECR1, NBN, OSGIN2 (C8orf1), RIPK2, TMEM64 (DFKZp762C1112)
+	8q22.1-22.2	99,007,325-100,014,623	C8orf47, NIPAL2, HRSP12, KCNS2, MATN2, POP1, RPL30, STK3
+	8q24.11	118,393,066-118,844,178	MED30 (THRAP6)
+	8q24.3	140,665,174-140,805,057	KCNK9
+	12p12.1	22,535,457-24,308,820	C2CD5 (KIAA0528), ETNK1, SOX5
+	12q12	40,876,491-42,688,707	ADAMTS20, TMEM117, IRAK4, PPHLN1, PRICKLE1, PUS7L (DKFZP434G1415), TWF1 (PTK9), YAF2, ZCRB1 (MADP-1)
+	13q13.3	37,635,092-38,496,395	UFM1, FREM2, STOML3, PROSER1
+	13q21.2	58,572,030-60,726,339	DIAPH3, TDRD3
+	13q22.1	72,583,695-73,033,999	-
+	13q22.2	74,725,315-74,918,459	TBC1D4
+	13q31.1	84,731,597-85,139,789	-
+	13q31.3	90,532,984-91,293,729	GPC5
+	13q33.1-33.2	103,314,852-104,473,863	-
+	20q13.13-13.2	47,868,388-49,291,771	KCNG1, ADNP, BCAS4, CEBPB, DPM1, FAM65C (C20orf175), MOCS3, PARD6B, PTPN1, RNF114 (ZNF313), SNAI1, SPATA2, TMEM189 (Kua), TMEM189-UBE2V1 (Kua-UEV), UBE2V1, SLC9A8
-	8p22	13,833,679-14,445,035	SGCZ
-	18p11.23	7,579,318-7,816,623	PTPRM
-	18p11.21	12,535,581-14,045,454	SPIRE1, PSMG2, CEP76, PTPN2, SEH1L, CEP192, LDLRAD4, FAM210A, RNMT, MC5R, MC2R
-	18q12.1	25,987,928-27,146,504	DSC3, DSC2, DSC1
-	18q23	71,882,399-74,199,087	ZNF516, FLJ44313, FLJ44881, ZNF236, MBP, GALR1
-	22q11.1-11.21	15,685,581-16,590,946	CECR2, GAB4, ATP6V1E1, BCL2L13, CECR1, CECR5, CECR6, IL17RA, SLC25A18, CECR3, CECR7, IGKV1-12
