# Supplementary material for: Loss of PTPRM Associates with the Pathogenic Development of Colorectal Adenoma-Carcinoma Sequence
Source: Sci Rep. 2015 Apr 24;5:9633. doi: 10.1038/srep09633 (PMC5386118; doi:10.1038/srep09633)
Supplement: Supplementary Information — Supporting information [file srep09633-s1.pdf]

Supporting information

Loss of *PTPRM* Associates with the Pathogenic Development of Colorectal Adenoma-Carcinoma Sequence

Putty-Reddy Sudhir[1], Shiu-Ting Lin[1], Chien Chia-Wen[1], Shung-Haur Yang[2], Anna Fen-Yau Li[3], Rai-Hua Lai[1], Mei-Jung Wang[1], Yuan-Tsong Chen[1], Chian-Feng Chen[4], Yuh-Shan Jou[1], and Jeou-Yuan Chen[1,5*]

[1]Institute of Biomedical Sciences, Academia Sinica, Taipei, Taiwan, ROC; Departments of [2]Surgery and [3]Pathology, Taipei Veterans General Hospital, Taiwan, ROC; [4]VYM Genome Research Center, Taiwan, ROC; [5]Institute of Genome Sciences, National Yang-Ming University, Taipei, Taiwan, ROC

Supplementary Table S1. Minimal common regions of CNAs associated with colon carcinomas identified by 100K oligonucleotide array analysis are listed. These minimal CNAs were identified by CNAG analysis and further confirmed by dCHIP analysis in at least two samples. The 19 gain regions identified in both adenomas and carcinomas by CNAG and dCHIP analyses are shown with asterisk. CN gains (+), CN loss (-), cytoband, physical position, and samples involving adenomas (A) and carcinomas (T) are shown.

		CNAG /dCHIP analysis	
	Cytoband	Minimal common region	Samples involved
+	6q16.1	94,211,682-94,229,212	10A, 11A, 12A
+	6q24.1-24.2	142,881,191-144,554,694	11A, 12A, 3T, 5T
+	6q25.3	155,789,931-158,108,265	11A, 12A, 3T, 5T
+	7p22.3-21.3	250,149-8,783,476	2A, 3A, 3T, 6T, 7T, 8T
+	7p21.3-21.2	12,294,465-13,254,072	2A, 3A, 2T, 3T, 6T, 7T, 8T
+	7p21.1	16,181,205-18,220,618	2A, 3A, 2T, 3T, 6T, 7T, 8T

+	7p21.1-14.2	19,732,775-36,908,719	2A, 3A, 2T, 3T, 6T, 7T, 8T
+	7p14.1-12.3	38,105,534-48,771,286	2A, 3A, 2T, 3T, 6T, 7T, 8T
+	7p12.3	49,309,682-49,610,949	2A, 3A, 10A, 11A, 14A, 2T, 3T, 6T, 7T, 8T
+	7p12.2-12.1	49,916,060-52,226,412	2A, 3A, 2T, 3T, 6T, 7T, 8T
+	7p12.1-11.1	53,498,522-57,653,125	2A, 3A, 2T, 3T, 6T, 7T, 8T
+	7q21.13-31.1	88,727,093-108,170,237	2A, 3A, 3T, 6T, 7T, 8T
+	7q31.2-31.31	114,825,957-120,500,140	2A, 3A, 3T, 6T, 7T, 8T
+	7q36.2-36.3	152,455,069-158,624,663	2A, 3A, 3T, 5T, 6T, 7T, 8T
+	8p23.3-23.2	2,131,936-2,354,297	11A, 13A, 14A
+	8q11.1-11.21	47,365,080-48,899,777	2T, 3T, 5T, 6T, 8T
+	8q11.21-11.23	52,263,997-53,403,770	11A, 12A, 2T, 5T, 6T, 8T
+	8q12.1	59,695,261-60,005,224	1T, 2T, 5T, 6T, 8T
+	8q12.2-12.3	61,926,630-63,034,569	1T, 2T, 3T, 5T, 6T, 8T
+	8q13.2	68,333,486-70,373,374	2T, 5T, 6T, 8T
+	8q13.3-21.11	73,254,861-74,383,960	2T, 3T, 5T, 6T, 8T
+	8q21.11	74,492,438-75,880,359	2T, 3T, 5T, 6T, 8T
+	8q21.12-21.13	80,134,969-80,660,427	11A, 12A, 2T, 3T, 5T, 6T, 8T
+	8q21.13	81,208,496-82,860,180	1T, 2T, 3T, 5T, 6T, 8T
+	8q21.13	83,311,297-83,415,682	2T, 3T, 5T, 6T, 8T
+	8q21.13	83,457,039-83,596,175	2T, 3T, 5T, 6T, 8T
+	8q21.2	85,946,905-86,526,160	2T, 5T, 6T, 8T
+	8q21.2-21.3	86,578,680-87,658,121	2T, 5T, 6T, 8T
+	8q21.3	88,669,709-89,287,326	2T, 5T, 6T, 8T
+	8q21.3	90,709,838-92,033,734*	11A, 12A, 2T, 3T, 5T, 6T, 8T
+	8q22.1	94,453,866-95,348,936	11A, 12A, 1T, 2T, 5T, 6T, 8T
+	8q22.1-22.2	99,007,325-100,014,623*	11A, 12A, 1T, 2T, 3T, 5T, 6T, 8T
+	8q22.2-22.3	101,557,726-104,458,292	11A, 12A, 1T, 2T, 5T, 6T, 8T
+	8q23.1	107,526,431-109,218,441	2T, 3T, 5T, 6T, 8T
+	8q23.1-23.3	110,122,659-112,917,443	2T, 5T, 6T, 8T
+	8q23.3	113,002,881-114,055,290	2T, 3T, 5T, 6T, 8T
+	8q23.3	114,346,369-115,097,126	2T, 3T, 5T, 6T, 8T
+	8q24.11	117,789,411-118,265,984	1T, 2T, 5T, 6T, 8T
+	8q24.11	118,393,066-118,844,178*	11A, 12A, 1T, 2T, 5T, 6T, 8T
+	8q24.11-24.12	119,005,983-119,333,613	11A, 12A, 1T, 2T, 5T, 6T, 8T
+	8q24.12	120,399,504-121,147,495	1T, 2T, 3T, 5T, 6T, 8T
+	8q24.13-24.21	124,731,100-129,166,049	1T, 2T, 3T, 5T, 6T, 8T
+	8q24.21	129,177,879-130,098,574	1T, 2T, 3T, 5T, 6T, 8T

+	8q24.21	130,413,367-131,108,691	1T, 2T, 3T, 5T, 6T, 8T
+	8q24.23	139,138,355-139,261,730	1T, 2T, 3T, 5T, 6T, 8T
+	8q24.3	140,538,976-140,640,767	9A, 10A, 11A, 12A, 1T, 2T, 3T, 5T, 6T, 8T
+	8q24.3	140,665,174-140,805,057*	9A, 10A, 11A, 12A, 1T, 2T, 3T, 5T, 6T, 8T
+	8q24.3	142,072,079-146,052,174	1T, 2T, 3T, 5T, 6T, 8T
+	12p12.1	22,535,457-24,308,820*	11A, 12A, 5T, 7T
+	12q12	40,876,491-42,688,707*	11A, 12A, 5T, 7T
+	12q12-13.11	43,002,077-46,023,714	5T, 7T
+	13q12.13-12.3	25,659,382-30,486,273*	3A, 12A, 1T, 2T, 3T, 5T
+	13q13.3	37,635,092-38,496,395*	3A, 12A, 2T, 3T, 5T
+	13q14.11-14.13	39,852,138-45,310,109*	3A, 12A, 1T, 2T, 3T, 5T
+	13q14.3-21.1	52,997,850-58,495,007*	3A, 12A, 2T, 3T, 5T
+	13q21.2	58,572,030-60,726,339*	3A, 12A, 2T, 3T, 5T
+	13q21.2-21.33	60,816,564-70,223,053*	3A, 12A, 2T, 3T, 5T
+	13q22.1	72,583,695-73,033,999*	3A, 12A, 1T, 2T, 3T, 5T, 6T
+	13q22.2	74,725,315-74,918,459*	3A, 12A, 1T, 2T, 3T, 5T, 6T
+	13q31.1	84,731,597,-85,139,789*	3A, 12A, 1T, 2T, 3T, 5T
+	13q31.3	90,532,984-91,293,729*	3A, 12A, 1T, 2T, 3T, 5T
+	13q32.1-32.3	95,724,865-100,190,749*	3A, 12A, 1T, 2T, 3T, 5T
+	13q33.1-33.2	103,314,852-104,473,863*	3A, 12A, 2T, 3T, 5T
+	15q21.1	44,155,507-44,569,827	10A, 11A, 12A
+	15q21.3	55,848,087-55,878,620	10A, 11A, 12A
+	15q22.2	61,049,905-61,052,297	10A, 11A, 12A
+	20p11.23-11.21	19,906,303-24,997,283	1T, 3T, 6T
+	20q11.21	29,309,964-30,949,712	1T, 2T, 3T, 5T, 6T
+	20q11.21-11.22	31,224,759-31,919,527	1T, 2T, 3T, 5T, 6T
+	20q11.22-11.23	31,946,293-36,910,448	1T, 2T, 3T, 5T, 6T
+	20q11.23-12	37,054,302-40,624,919	1T, 2T, 3T, 5T, 6T
+	20q13.13	46,296,791-47,764,927	9A, 12A, 1T, 2T, 3T, 5T, 6T
+	20q13.13-13.2	47,868,388-49,291,771*	9A, 12A, 1T, 2T, 3T, 5T, 6T
+	20q13.2	49,387,119-50,001,018	9A, 12A, 1T, 2T, 3T, 5T, 6T
+	20q13.2-13.31	50,345,902-53,643,287	1T, 2T, 3T, 5T, 6T
+	20q13.31	53,665,970-54,804,260	1T, 2T, 3T, 5T, 6T
+	20q13.31-13.32	54,946,661-55,465,932	1T, 2T, 3T, 5T, 6T
+	20q13.32	55,526,741-56,740,860	1T, 2T, 3T, 5T, 6T
+	20q13.32	57,034,050-57,352,911	1T, 2T, 3T, 5T, 6T

+	20q13.33	59,832,199-59,834,230	9A, 10A, 12A, 1T, 2T, 3T, 5T, 6T
-	1p36.23-36.21	7,572,245-14,788,323	5T, 6T
-	4q34.3	179,246,247-183,373,475	3T, 6T
-	5q21.3-22.3	107,430,837-113,333,210	2A, 11A, 14A, 3T, 6T
-	8p22	13,833,679-14,445,035	2T, 5T, 6T
-	15q21.1-21.2	44,973,231-48,074,273	1T, 3T, 5T
-	15q21.3-22.1	51,447,773-57,096,765	1T, 3T, 5T
-	17p13.1-12	8,791,713-13,954,919	1T, 3T, 5T, 6T
-	18p11.32-11.31	867,772-7,054,691	1T, 3T, 5T, 6T
-	18p11.23	7,579,318-7,816,623	1T, 3T, 5T, 6T
-	18p11.21	12,535,581-14,045,454	1T, 3T, 5T, 6T
-	18q12.1	25,987,928-27,146,504	1T, 3T, 5T, 6T
-	18q12.2-12.3	32,830,788-40,891,788	1T, 3T, 5T, 6T
-	18q21.1-21.2	46,349,352-50,885,624	1T, 2T, 3T, 5T, 6T
-	18q21.33-23	58,072,967-71,547,371	1T, 2T, 3T, 5T, 6T
-	18q23	71,882,399-74,199,087	1T, 2T, 3T, 5T, 6T
-	22q11.1-11.21	15,685,581-16,590,946	3T, 5T

Supplementary Figures

a

			**# of Samples**		
**Array Type**	**No.**	**Acc. No.**	**Normal**	**Adenoma**	**Tumor**
		**NCBI GEO**			
U 133 plus 2.0	1	GSE4107	10		12
	2	GSE4183	8	15	15
	3	GSE5206	5		100
	4	GSE8671	32	32	
U133A	5	GSE2138			20
	6	GSE4045			37
	7	GSE4554			84
	8	GSE7208			59
		**SMD**			
cDNA	9	Unpublished*	9	11	52
		**Total**	64	58	379

b

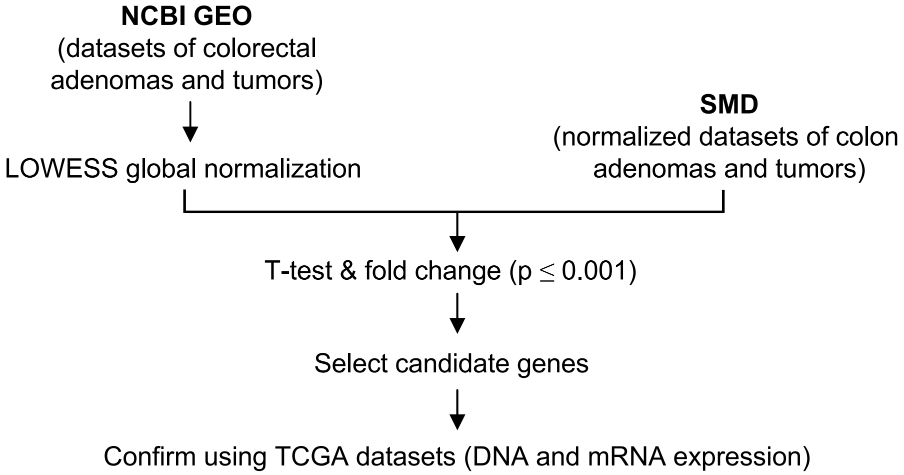

Supplementary Fig. S1. *In silico* analysis of candidate genes associated with CNAs in colon adenomas and carcinomas. (a) Relative expression of candidate genes associated with CNAs in colorectal adenomas and carcinomas was obtained from 9 datasets available on NCBI's GEO and SMD databases, consisting samples corresponding to 64 non-tumorous, 58 adenomas and 379 tumor samples. (b) Flow chart of candidate gene identification. The eight datasets obtained from NCBI's GEO were normalized by LOWESS analysis. The gene expression levels in colorectal adenomas and tumors relative to normal samples were determined by means of t-test significance ($p \leq 0.001$). The selected candidate genes were further confirmed for DNA and mRNA copy numbers using TCGA colorectal DNA and expression datasets. *Unpublished data was kindly provided by Chen Xin et al., Department of Biopharmaceutical Sciences, School of Pharmacy, University of California, San Francisco.

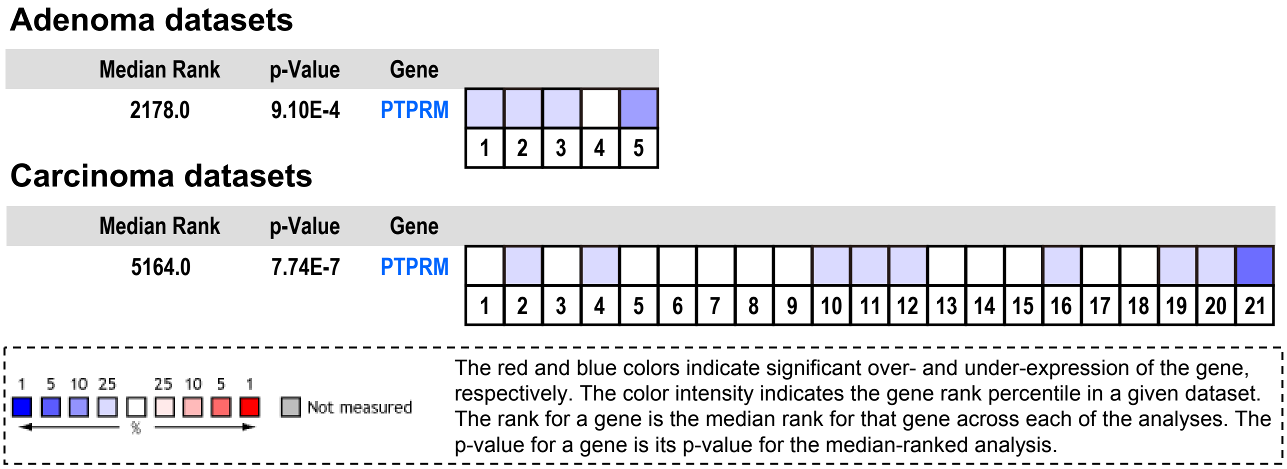

Adenoma datasets

1. Colorectal Adenoma Epithelia vs. Normal
Gaspar Colon, Am J Pathol, 2008
2. Colon Adenoma vs. Normal
Sabates-Bellver Colon, Mol Cancer Res, 2007
3. Rectal Adenoma vs. Normal
Sabates-Bellver Colon, Mol Cancer Res, 2007
4. Colon Adenoma Epithelia vs. Normal
Skrzypczak Colorectal 2, PLoS One, 2010
5. Colon Adenoma vs. Normal
Skrzypczak Colorectal 2, PLoS One, 2010

Carcinoma datasets

1. Rectal Adenocarcinoma vs. Normal
Gaedcke Colorectal, Genes Chromosomes Cancer, 2010
2. Colorectal Carcinoma vs. Normal
Graudens Colon, Genome Biol, 2006
3. Colorectal Carcinoma vs. Normal
Hong Colorectal, Clin Exp Metastasis, 2010
4. Cecum Adenocarcinoma vs. Normal
Kaiser Colon, Genome Biol, 2007
5. Colon Adenocarcinoma vs. Normal
Kaiser Colon, Genome Biol, 2007
6. Colon Mucinous Adenocarcinoma vs. Normal
Kaiser Colon, Genome Biol, 2007
7. Rectal Adenocarcinoma vs. Normal
Kaiser Colon, Genome Biol, 2007
8. Rectal Mucinous Adenocarcinoma vs. Normal
Kaiser Colon, Genome Biol, 2007
9. Rectosigmoid Adenocarcinoma vs. Normal
Kaiser Colon, Genome Biol, 2007
10. Colon Adenocarcinoma vs. Normal
Ki Colon, Int J Cancer, 2007
11. Colon Adenocarcinoma vs. Normal
Notterman Colon, Cancer Res, 2001
12. Colorectal Adenocarcinoma vs. Normal
Skrzypczak Colorectal, PLoS One, 2010
13. Colorectal Carcinoma vs. Normal
Skrzypczak Colorectal, PLoS One, 2010
14. Colon Carcinoma Epithelia vs. Normal
Skrzypczak Colorectal 2, PLoS One, 2010
15. Colon Carcinoma vs. Normal
Skrzypczak Colorectal 2, PLoS One, 2010
16. Cecum Adenocarcinoma vs. Normal
TCGA Colorectal, 2011
17. Colon Adenocarcinoma vs. Normal
TCGA Colorectal, 2011
18. Colon Mucinous Adenocarcinoma vs. Normal
TCGA Colorectal, 2011
19. Rectal Adenocarcinoma vs. Normal
TCGA Colorectal, 2011
20. Rectal Mucinous Adenocarcinoma vs. Normal
TCGA Colorectal, 2011
21. Rectosigmoid Adenocarcinoma vs. Normal
TCGA Colorectal, 2011

Supplementary Fig. S2. Loss of PTPRM is a frequent event in colorectal adenomas and carcinomas. The data sets related to colorectal adenoma and carcinoma samples were compared with normal samples by means of Oncomine database. The 26 data sets (five adenoma and 21 carcinoma sets) used in this analysis were listed. The significance (p-value) of PTPRM down-regulation observed in adenoma and carcinoma datasets in Oncomine analysis is shown.

Colon adenocarcinoma

Gene Set	Source	Size	ES	NES	NOM p-val	FDR q-val
NECTIN PATHWAY	PID	30	-0.338	-0.989	0.478	0.422
ADHERENS JUNCTION	KEGG	73	-0.348	-1.182	0.271	0.346
CELL ADHESION MOLECULES CAMS	KEGG	125	-0.359	-1.2	0.268	0.651

Rectal adenocarcinoma

Gene Set	Source	Size	ES	NES	NOM p-val	FDR q-val
NECTIN PATHWAY	PID	30	-0.348	-0.997	0.432	0.396
ADHERENS JUNCTION	KEGG	73	-0.473	-1.538	0.057	0.058
CELL ADHESION MOLECULES CAMS	KEGG	125	-0.465	-1.6	0.046	0.093

Supplementary Fig. S3. Gene set enrichment analysis (GSEA) analysis results. Three PTPRM-associated pathways were downloaded from molecular signature database of GSEA. The colon and rectal adenocarcinoma expression data sets were downloaded from TCGA database. The GSEA analysis of TCGA datasets was performed using default parameters to identify the significance of the deregulation of PTPRM-associated pathways. ES, enrichment score; NES, normalized enrichment score.
